# The Effect of Complex Forming Cations on the Thermophysical Properties of Beryllium and Uranium Fluoride Salts for Nuclear Reactor Applications

**DOI:** 10.1021/acsphyschemau.5c00043

**Published:** 2025-10-16

**Authors:** D. Nathanael Gardner, Maximilien Denis, Michael Borrello, Christian Sclafani, Raluca O. Scarlat

**Affiliations:** † Department of Nuclear Engineering, 1438 University of California, Berkeley, California 94720, United States

**Keywords:** Molten Salt, Beryllium, Uranium, Density, Viscosity, Nuclear Fuel, Nuclear Reactor

## Abstract

Molten beryllium and uranium containing fluoride salts, such as NaF-BeF2-UF4-ZrF4 and NaF-BeF2 are examples of fuel solvent and heat transfer salts used in molten salt reactor designs. To observe the behavior of these salts and to ascertain the mechanisms behind the formation of ionic complexes present in their molten state, this work used high temperature rheology and hydrostatic density methods to measure thermophysical properties. Similar to modeling literature, two regions of viscosity were identified: one below 60 molar percentage of complex forming cations, where it is hypothesized that viscosity is driven by the diffusion of small ionic fragments, and one above where it is hypothesized the degree of polymerization of the complexing cation and network formation drives the increase in viscosity.

## Graphical abstract

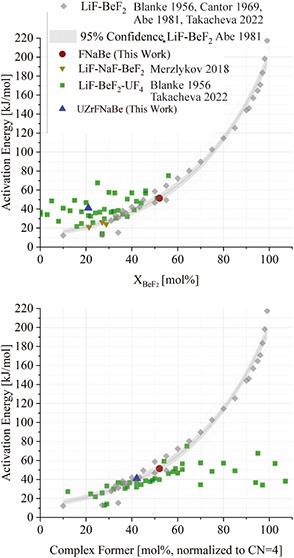

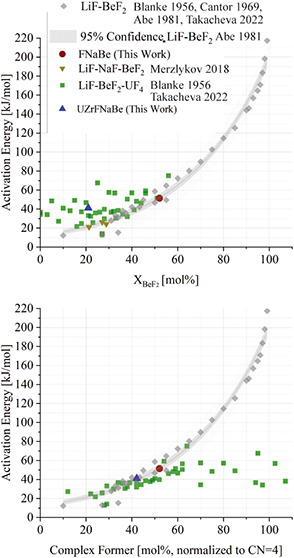

## 1 Introduction and Background

Density and viscosity are two of the thermophysical properties of fluoride salts, whose characterization is useful for molten salt reactor (MSR) applications. For example, in molten salt reactor designs that incorporate natural circulation salt loops, thermal expansivity, density, and viscosity are necessary to design the required loop geometry for natural circulation and passive heat removal. Density and viscosity also are essential to calculating pumping power required for salt flow, predicting the migration of gaseous fission products in the reactor core, and designing for effective heat transfer in the heat exchanger.^1–3^ NaF-BeF_2_–UF_4_–ZrF_4_ denoted UZrFNaBe, and NaF-BeF_2_ denoted FNaBe, are two candidate salts for molten salt reactors as a fuel and heat transfer fluid, respectively.

During molten salt reactor operations, in addition to transients such as temperature and salt flow rate due to the fission process, elements across the periodic table are present in the salt system. Understanding of the mechanisms of complex formation in uranium and beryllium containing fluoride salts and its effect on density and viscosity allows for more accurate modeling of molten salt thermophysical property behavior at various temperature conditions as well as better prediction of the effect of fission and corrosion products over the reactor lifetime.^4–6^

Beryllium fluoride containing molten salt systems has been studied for decades through a variety of methods. In 1969, a polymer model was proposed describing the oligomer forming behavior of BeF_2_ containing molten salts and hypothesized that increasing the BeF_2_ content in a melt increased the number and length of long polymer chains present which are formed from BeF_4_^2–^ linkages through corner sharing fluorines.^7^ Thermophysical property measurements such as viscosity have been used to demonstrate how increasing the BeF_2_ content in a LiF-BeF_2_ melt increases the viscosity and activation energy of viscosity due to network formation.^8^ Viscosity has also been used to observe how in BeF_2,_ a tetrahedrally linked network-forming liquid, the molten structure is weakened by the addition of components such as LiF which cause the severing of bridging fluorines.^9^ This structural network-breaking behavior has also been noted to be caused by the addition of CeF_3_ in LiF-NaF-BeF_2_ melts.^10^ X-ray and neutron diffraction studies as well as *ab initio* and neural network molecular dynamic simulations have been used to confirm the presence of these network species and measure the coordination number and polymer chain length of fluoroberyllate complexes.^11^ This relationship between properties and complex ion structure is also present when the weakly coordinating cation Li^+^ is instead Na^+^ as is the case in NaF-BeF_2_ melts.^12^

Similarly, experimental and modeling studies have shown that uranium-containing molten fluoride salts have been found to form polymerized networks of corner, face, and edge sharing complexes.^13^ Coordination numbers for beryllium and uranium in fluoride salts have been measured and simulated to be around 4 and 8 respectively.^11,13,14^ Zirconium fluoride melts have also been studied through experimental and simulation techniques and measured to have a coordination number ranging from 6 to 9.^15,16^

Molecular dynamics computer simulation has identified two regions of viscosity mechanisms in LiF-BeF_2_ melts.^4^ At low BeF_2_ content below 50 molar percentage, viscosity is primarily driven by the diffusion of ionic species such as BeF_4_^2–^ formed by the 4-fold coordination of beryllium, while at high BeF_2_ content the main driver of viscosity is the structural relaxation of beryllium oligomer networks as the length of the polymeric chains increase.^4^ UF_4_ melts have been modeled to show similar behavior to fluoroberyllate melts;^13^ it exists primarily as UF_8_^4–^ ionic species at low uranium content below 20 molar percentage, and has network forming character at high uranium content.

The goal of this work is to use thermophysical properties, namely, viscosity, density, and thermal expansivity, to probe the structure NaF-BeF_2_–UF_4‑_ZrF_4_ and NaF-BeF_2_ to compare the mechanisms of uranium and beryllium network forming behavior. Comparing density and thermal expansivity values with literature and experimentally providing currently unpublished values of viscosity for potential advanced reactor fuel and coolant salts provides data pertinent to molten salt reactor applications and betters understanding of the effect of solvated fuel on the thermophysical properties of salt melts.

## 2 Materials and Methods

### 2.1 Elemental Analysis and Composition Determination

Compositional measurement was completed using dilute 20 M nitric acid in a CEM SP-Discover 80 Microwave Digester System for salt digestion and a PerkinElmer 5300 DV Inductively Coupled Plasma – Optical Emission Spectrometer (ICP-OES) for elemental analysis. Percent recovery and digestion efficiency was calculated from the ICP-OES measured intensity of the initial masses of approximately 0.05 g of sample digested compared to the calibration curve created using Inorganic Ventures elemental analysis standards.

### 2.2 Density Measurements

Density measurements were performed by using the hydrostatic method. This method is based on the principle of Archimedes: a body fully immersed in a fluid experiences a force equal to the weight of the displaced fluid. The setup shown in Figure 1 was placed in an argon glovebox (LC Technology Solutions Inc.) with an atmosphere of argon (AirGas cylinder 99.999% purity). Pressure was kept between P_atm_-0.2 mbar and P_atm_-0.1 mbar, and oxygen and moisture levels were kept below 5 and 0.1 ppm, respectively. The volume of the 316 stainless-steel bobber (designed and machined at UC Berkeley) was calibrated using Cargille Laboratories NIST traceable organic series fluids of 0.800 and 3.310 g/cm^3^ density. The bobber was then suspended from the hook of a GH-252 analytical scale (A&D Company), with a precision of 0.1 mg, using a 0.51 mm diameter nickel-chrome wire (WIREOPTIM). Underneath the bobber and scale, a glassy carbon crucible measuring 50 mm in diameter and 85 mm in height (SPI Supplies) containing the salt and a type N thermocouple (OMEGA, TJ36-NNIN-116U-18-SB-SMPW-M) was located inside of a CFRC-36/115-A vertical tube furnace (WATLOW) and placed on a scissor jack. The furnace and thermocouple were connected to a Platinum Series proportional–integral–derivative (PID) controller (OMEGA, part# CS8DPT) to set a stable temperature for each experiment (P = 8.8; I = 0.001; D 336.00).

**Figure 1:**
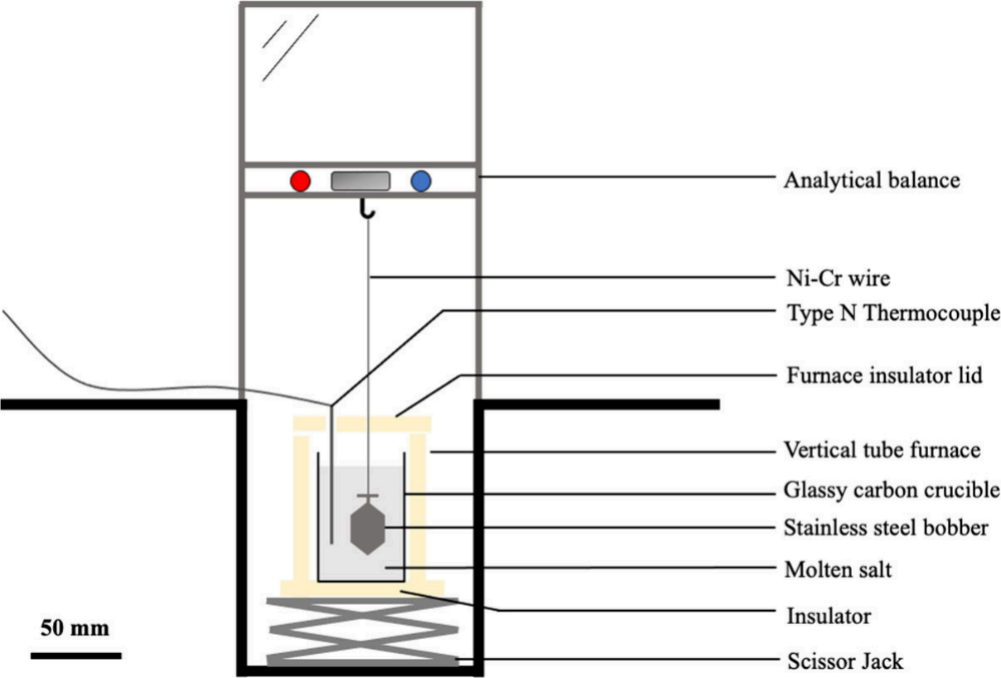
Scheme of the hydrostatic method setup.

After the bobber was hung, the scale was tared, the salt was melted, and the bobber was immersed into the molten salt using the scissor jack to lift the furnace assembly. After complete immersion of the bobber into the molten salt was confirmed visually, the value measured by the scale corresponded to the difference of weight Δ*M*. Δ*M* measurements were made with NIST-traceable density calibration fluids (0.8000 and 0.3100 g/cm3) at 25 °C and with molten salt at high temperature.

After the immersion of the bobber, an insulating lid was placed on top of the furnace. Δ*M* was then recorded every 10 s for 15 min (90 points) using the RsCom WinCT software (A&D Company). Average Δ*M* and standard deviation were calculated on 90 points at each temperature T. After each experiment, salt was left to cool to ambient temperature by using the glovebox cooling fans and heat exchanger. Frozen salt was then removed from the crucible and stored in a plastic container in the glovebox. Figure 2 gives density benchmarking results for LiF-BeF_2_ (FLiBe) and thermocouple cooling curves from this setup.

**Figure 2:**
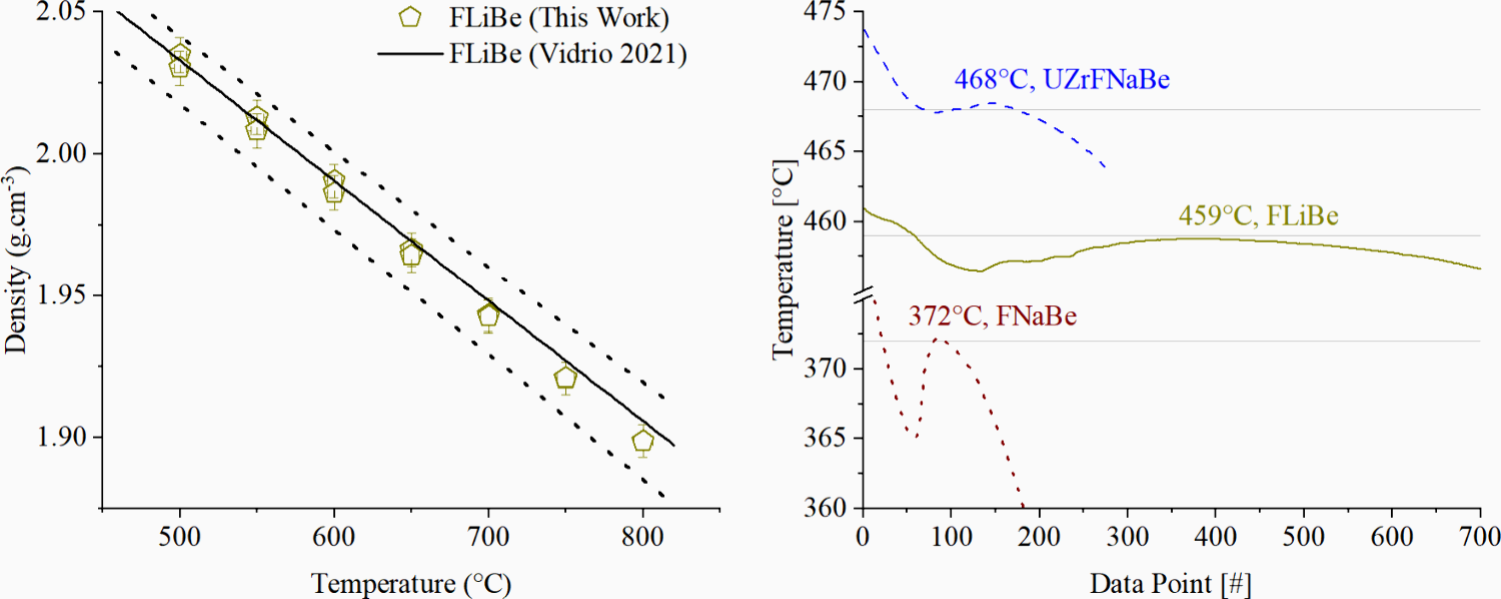
(Left) Density benchmarking against previously reported FLiBe.^17^ (Right) Melting points of the studied salts measured by freezing the type N thermocouple in the salt prior to the density measurement.

The temperature measurements reported by the thermocouple (TC) have an error of ± 2.2 °C or 0.75% of the reported value in °C, depending on which value is larger; thus, for the temperature measurements in this study, each measurement contains an implicit 0.75% uncertainty which is propagated across all three independent temperature measurements.

Validation of TC readings was performed by comparison to the melting point of FLiBe observed during salt cooling curves, where the TC is frozen in the salt. We believe that the same TC was used for FNaBe and UZrFNaBe density vs temperature measurements; though we note that chain of custody quality assurance was not applied to the TC, so we cannot exclude the possibility that other oven users replaced the TC in-between the two measurements.

To ensure thermal equilibration, the system was held at the targeted temperature for 40 min before measuring each data point to allow the system to fully reach equilibrium. The temperature inaccuracy due to thermocouple lag is negligible for this study: for the thermocouple used here, the reported time constant is approximately 6 s,^18^ and the difference in temperature of the environment from the temperature measured during a 5 °C/min ramp with our thermocouple would be 0.5 °C which is within the reported thermocouple error.^19^

Density is computed from the measured Δ*M* at temperature, and the volume at temperature, computed from the calibrated bobber volume at 25 °C and the computed thermal expansion of stainless steel 316, α_*SS*316_ ^20^ (see Supplementary Data for the temperature-dependent α_*SS*316_). Surface tension correction^17^ on the mass difference is negligible (<0.003%) for the size of the bobber used in this experiment (100.7145g stainless steel 316). Thus, the density of the salt is computed as follows:

$$\begin{array}{l} \rho_{salt}\left(T\right)=\frac{\Delta M_{salt}\left(T\right)}{V\left(T\right)}\\ V_{25{}^{\circ}C}=\frac{\Delta M_{NIST}}{\rho_{NIST}}\\ V\left(T\right)=V_{25{}^{\circ}C}\left[1+3\alpha_{SS316}\left(T-T_{25{}^{\circ}C}\right)\right]\\ \rho_{salt}\left(T\right)=\frac{\Delta M_{salt}\left(T\right)}{V\left(T\right)} \end{array}$$ 1

The error contributions to the measured salt density were: 0.3% due to variability between *V*_25°*C*_ calibrations with the two NIST-certified density fluids; 0.3% due to uncertainty in thermal expansion of the stainless steel bobber; and between 0.04% to 0.13% due to Δ*M*_*salt*_ measurement noise over the measurements time, once thermally equilibrated (see Supplementary Data for error propagation).

### 2.3 Viscosity

Viscosity experiments were performed using an Anton Paar Modular Compact Rheometer (MCR) 702e. In this experiment, the rotational viscosity method was used, utilizing a parallel plate measurement setup. For the rotational method, the shear stress τ (tau) was found by measuring the force of the salt’s resistance to flow as torque and dividing by the surface area of the parallel plates. Then, using a known and preset shear rate $\frac{dv}{dt}$ or γ (gamma), recorded by dividing the rotational velocity of the upper parallel plate by the measuring gap height between the two plates, the dynamic viscosity μ (mu) was calculated by dividing the shear stress by the shear rate (eqs 2–5). Shear rate was kept at 50 s^–1^ in all experiments; this value was the instrument vendor’s recommendation for this plate diameter. The measuring gap height was kept at a constant value of 1 mm for all measurements.

$$\tau =\mu \frac{dv}{dt}$$ 2

$$\tau \left(Shear\;Stress\right)=\frac{Force\;\left(F\right)}{Area\;\left(A\right)}$$ 3

$$\frac{dv}{dt}\left(Shear\;Rate\right)=\frac{Velocity\;\left(v\right)}{Height\;\left(h\right)}$$ 4

The measuring accessories used in this work were from Anton Paar Instruments. The upper and lower parallel plates had a diameter of 40 mm and are made from 316 stainless steel, while the shafts were made from Alloy 625 Inconel. The lower plate can be seen in Figure 3. The same plate set was used for all measurements, and between salt samples, the salt is scraped off and then sonicated in water until no residue is visible and then wiped dry with Kimwipes; if the plates contain debris or are not flat and smooth, then viscosity measurements will be incorrect.

**Figure 3:**
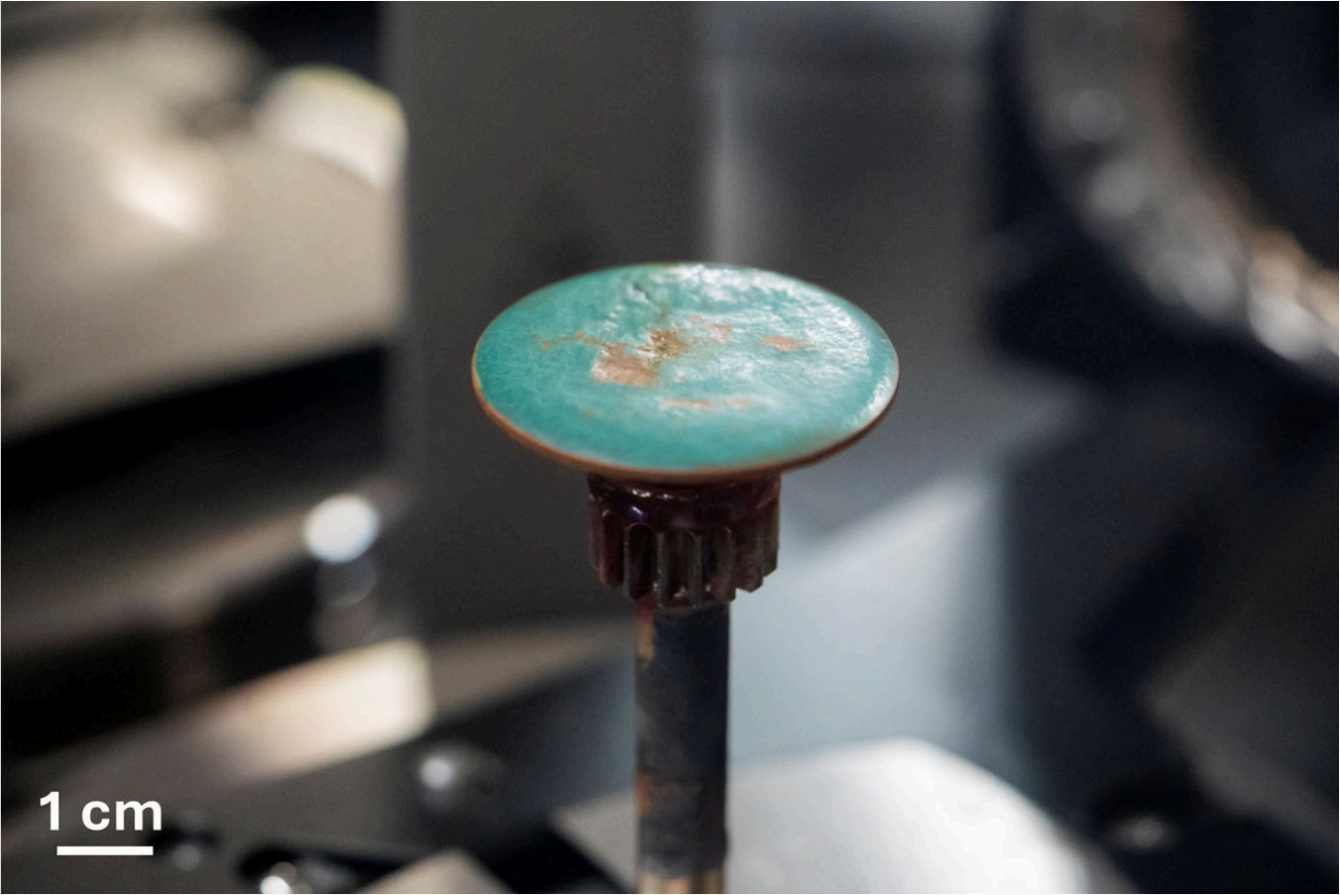
UZrFNaBe cooling on the lower parallel measuring plate after viscosity measurement. (Adam Lau/University of California Berkeley Engineering.)

The salt sample is loaded at room temperature, and then the clam-shell oven is closed and brought to temperature. The plates are brought to a distance of 1 mm after the salt melts, and then the oven is opened while the salt is melted, to verify that no surface area of the measuring plate remained uncovered by the salt; excess sample coming off the plate is scraped with the scraper supplied by Anton-Paar. The oven is then closed and allowed to equilibrate at the temperature set-point for 2 min, and then the viscosity measurement begins. Temperature is then ramped at a constant rate of 2.3 °C/min; if temperature ramp rates are too high, thermal equilibration is not achieved and viscosity readings are incorrect. Supplementary data also includes a 15 min temperature hold example for one of the samples, which illustrates viscosity variability with an isothermal hold, which in this study is lower than sample-to-sample variability. Figure 4 shows LiF_2_BeF_4_ (FLiBe) data, benchmarked against prior literature data for this melt.

**Figure 4:**
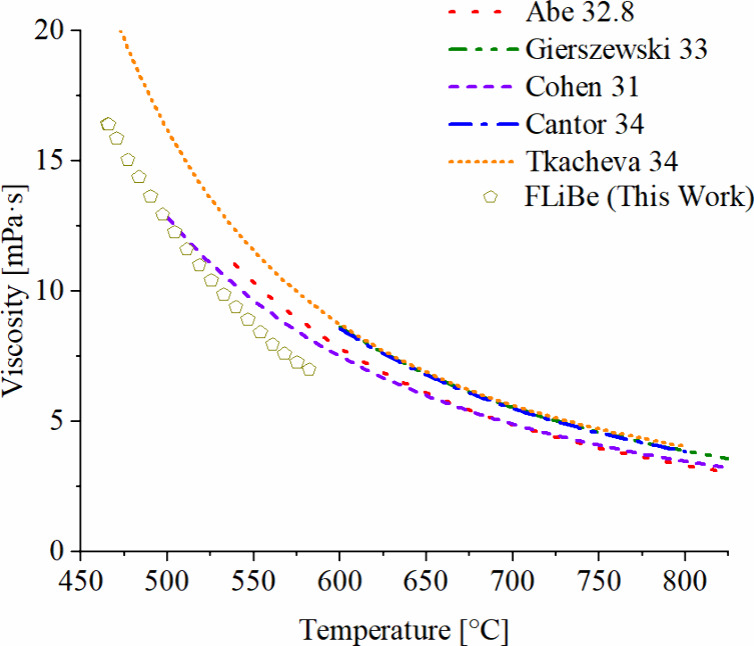
Viscosity benchmarking against prior FLiBe literature. ***x***_***BeF*_2_**_is indicated in the legend, for each reference; the expected *x*_*BeF*_2__ for this work is 33%.^8,21–25^

To extract the activation energy of the viscosity of the salts measured in this work, the viscosity Arrhenius equation was used (eq 5). This equation, slightly different from the typical Arrhenius equation due to the absence of a negative sign, is shown in eq 5 where *E*_a_ is the activation energy of viscosity, A is the pre-exponential constant, T is the temperature in K, and R is the universal gas constant.

$$\ln\left(\mu \right)=\ln\left(A\right)+\frac{E_{a}}{RT}$$ 5

### 2.4 Sample Preparation and Handling

Salt storage, sample preparation, and experimentation took place inside an argon glovebox with regulated oxygen and moisture levels. During salt storage, handling, and experimentation oxygen and moisture levels were kept below 5 and 0.1 ppm, respectively. As radioactive depleted uranium and toxic beryllium are both health hazards, regular monitoring was completed to ensure that levels remained below the levels released by the OSHA free release levels. Beryllium monitoring was completed through quarterly swiping of outer glovebox gloves, glovebox entrances, and general lab areas. Swipes were then sent out for digestion and analysis according to the OSHA ID-125G method. Of the 90 collected swipes over the data collection period, five were below 0.03 μg/100 cm^2^ and 85 of them were below detection (<0.0071 μg/100 cm^2^). In addition, air monitoring was performed during glovebox maintenance operations, and all 37 results were below detection (<0.04 μg/m3 or lower, depending on air sampling volume for each sample). During glovebox maintenance operations, full-face or half-face respirators were worn with P100 respirator cartridges. For radiation monitoring, finger ring thermos-luminescent dosimeters were worn during rad salt handling and experimentation. Laboratory radiation monitoring was conducted using a Ludlum Model 26 Integrated Frisker, for in-glovebox surveys, and a ThermoFisher Scientific Radey B20 along with swipes analyzed by PerkinElmer Tri Carb 290 TR Liquid Scintillation Analyzer for general lab surveys.

## 3 Results

### 3.1 Elemental Analysis and Melting Point

Results from the ICP-OES elemental analysis of FNaBe and UZrFNaBe are given in molar percentages in Table 1. The UZrFNaBe salt sample measured in this work is from the same sample batch as ref 26, and the target composition of the originally prepared batch of salt was 72:16.5:11:0.5 NaF-BeF_2_–UF_4_–ZrF_4_. Uncertainty was calculated on the UZrFNaBe sample using the error from volume additions during dilution and on the FNaBe sample using the propagation of the volume addition error and the standard deviation of the elemental analysis results.

**Table 1:** 

Sample	Digestion Efficiency (%)	X_NaF_	X_BeF2_	X_UF4_	X_ZrF4_	X_GdF3_
FNaBe	93(2)	0.48(1)	0.52(1)			
UZrFNaBe	99	0.68(3)	0.21(1)	0.102(5)	0.0044(2)	0.00040(2)

### 3.2 Density

UF_4_ and BeF_2_ species have very different estimated molar volumes, reflecting the different ionic radii of the cations (0.27 Å for Be^2+^ and 1 Å for U^4+^ in ionic crystals).^27^ Density values, including propagated uncertainty collected in this work using the hydrostatic method on the FNaBe and UZrFNaBe salts, are shown in Figure 5 and compared against experimental literature values for similar composition salts; melting points measured by the OMEGA type N thermocouples used in this work are also shown. Ideal density calculated using molar volume additivity was calculated based on two different sources for the molar volume of NaF and BeF_2_ single component melts: empirically estimated molar volumes^28^ and high temperature experimental data;^29^ other reported values of molar volumes and densities for NaF and BeF_2_ can be found in ref 30.

**Figure 5:**
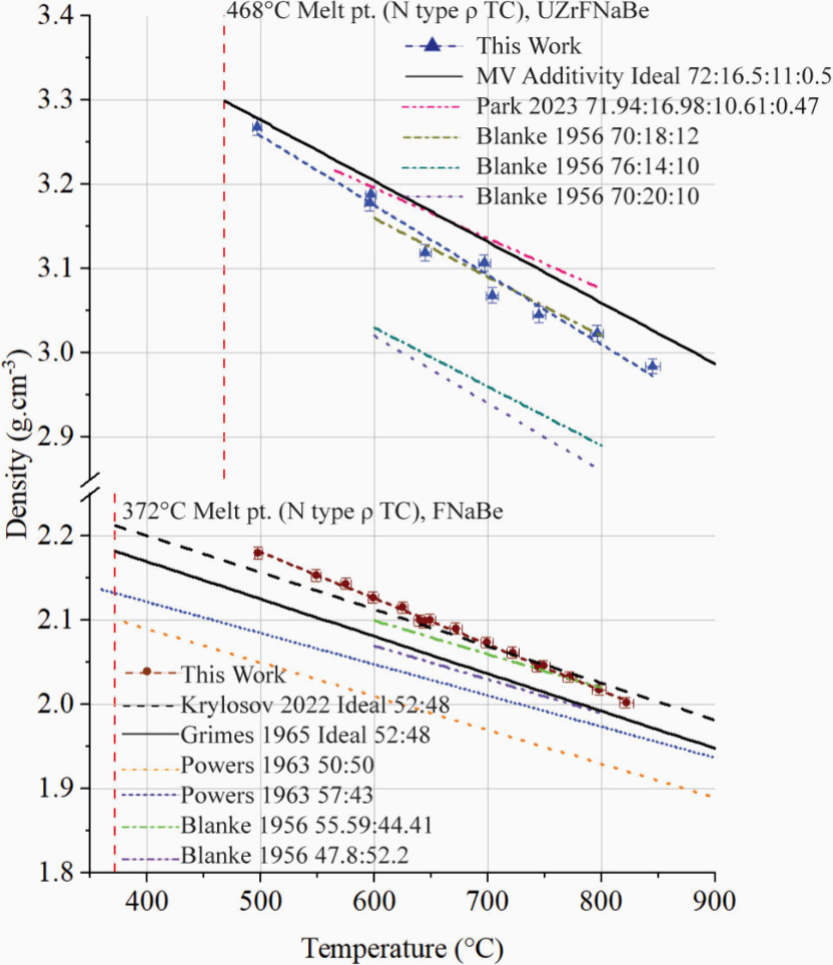
Densities of FNaBe and UZrFNaBe salts measured using the hydrostatic method, compared to literature values of similar composition salts.^26,31,32^ Comparison is limited to experimental work only and does not include modeling predictions. Molar compositions are given in the caption for BeF2:NaF:UF4:ZrF4 and BeF2:NaF.

A linear fit for the density of FNaBe and UZrFNaBe was calculated by performing a linear regression on the temperature dependent density data including propagated error for temperature and density using OriginPro v.10.0.0.154:

ρFNaBe[kgm3]=2454(13)−0.55(2)×T[C°],⁣forxBeF2=0.52(1),372to820C° 6

ρUZrFNaBe[kgm3]=3670(30)−0.83(5)×T[C°],⁣forxBeF2=0.21(1),xNaF=0.68(3)xUF4=0.102(5)xZrF4=0.0044(2),480to820C° 7

Thermal expansivity was calculated from the slope of the collected density values versus the temperature at which they were measured. Results are listed in Figure 6.

**Figure 6:**
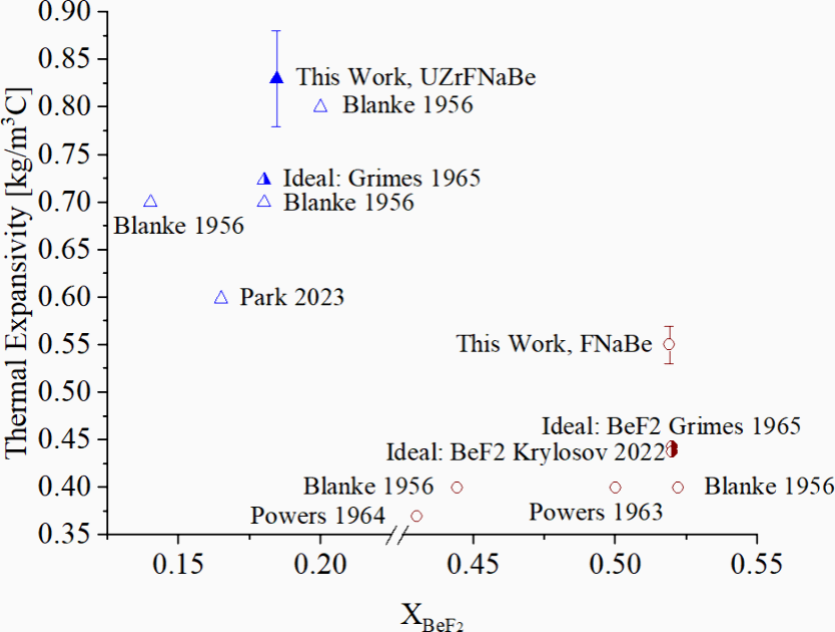
Thermal expansivities of FNaBe and UZrFNaBe measured in this work, compared with thermal expansivities from literature.^16,29,32^

### 3.3 Viscosity

#### 3.3.1 Temperature Dependence

Viscosity was measured in range from 400–600 and 490–600 °C for the FNaBe and UZrFNaBe salts, respectively. Data was collected in triplicate with three separate samples of each salt being taken from the bulk container of fresh salt. Uncertainty was quantified using the standard deviation of the three experiments. The resulting measurements are listed in Figure 7. Raw data, including viscosity, shear stress, torque, time, and temperature, for all viscosity runs are located in Supporting Information.

**Figure 7:**
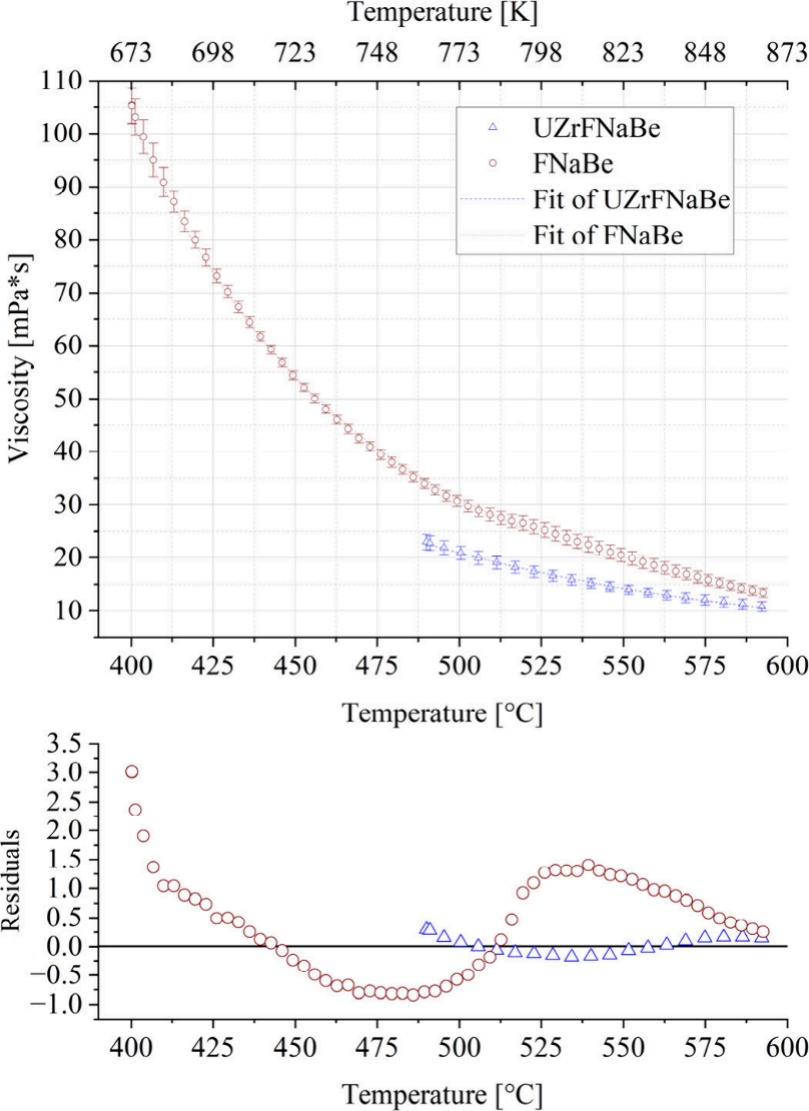
Viscosity of FNaBe and UZrFNaBe measured in this work using a 40 mm parallel plate rotational viscometer at a 50 s^–1^ shear rate. Error bar is standard deviation across three runs on three different samples from the same salt batch.

#### 3.3.2 Activation Energy

Temperature dependent viscosity values were collected for both salts and fit to eq 10. The resulting fitted lines are shown in Figure 8. Then using OriginPro, a linear fit was applied to extract the measured activation energy of viscosity in the form of the slope and pre-exponential constant A in the form of the y-intercept. Values for the eq 8 parameters *E*_a_ and A are located in Table 2.

**Table 2:** Activation Energy of Viscosity and Pre-exponential Constant Values Calculated for the Viscosity Measured in This Work

Sample Name	Temperature Range [°C]	A [mPa-s]	*E*_a_ [J/mol]
**FNaBe**	400–600	0.0103(8)	51,500(500)
	400–510	0.0071(9)	53,700(700)
	520–600	0.007(4)	55,000(5,000)
**UZrFNaBe**	490–600	0.05(2)	41,000(2,000)

**Figure 8:**
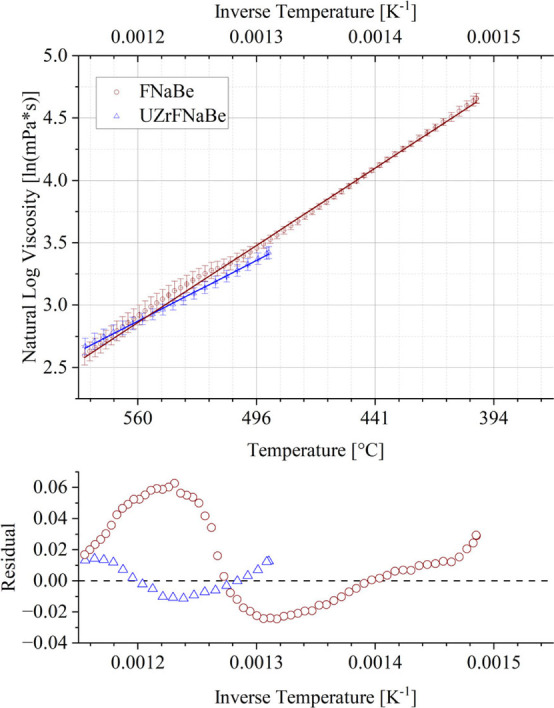
Activation energy of viscosity calculated of FNaBe and UZrFNaBe measured in this work using a 40 mm parallel plate rotational viscometer at 50 s^–1^ shear rate. Error bar is the standard deviation across three runs on three different samples from the same salt batch.

## 4 Discussion

### 4.1 Variations in Molar Volume Additivity Calculation

The thermal expansion coefficient, β, is expressed as follows from linear density functions:

8 ρFNaBe[kgm3]=2454(13)·(1−0.0224(8)[%C°]·T[C°]),xBeF2=0.52(1),372to820C°

9 ρUZrFNaBe[kgm3]=3670(30)·(1−0.0226(14)[%C°]·T[C°]),xBeF2=0.21(1)xNaF=0.68(3)xUF4=0.102(5)xZrF4=0.0044(2),488to820C°

Both the thermal expansion coefficient and the thermal expansivity *ρβ* measured in this work for FNaBe and UZrFNaBe are higher than the literature values (see Figure 6). The values predicted by the molar volume additivity model (Figure 6) are higher than prior literature values and lower than the values measured here.

Two linearity assumptions are possible for these single-component salts: either molar volume vs temperature is linear, or the density versus temperature is linear. Using molar volume additivity with these two assumptions arrives at eqs 8 and 9, respectively, for the temperature and composition dependence of the NaF-BeF_2_ binary system. By taking the partial derivative with respect to temperature, ${\left(\frac{\delta }{\delta T}\right)}_{x_{BeF_{2}}}$ , the slope of the ideal density (i.e., the thermal expansivity) was computed, which remains a function of temperature and composition and is illustrated in Figure 9 (see equation in Supporting Information^33,34^ ).

$$\rho_{NaF-BeF_{2},vlinearity}\left(x_{BeF_{2}},T\right)=\frac{x_{BeF2}{MW}_{BeF2}+\left(1-x_{BeF2}\right){MW}_{NaF}}{x_{BeF2}\left(0.004\times T+21.2\right)+\left(1-x_{BeF2}\right)\left(0.0056\times T+15.72\right)}$$ 10

$$\rho_{NaF-BeF_{2},\rho linearity}\left(x_{BeF_{2}},T\right)=\frac{x_{BeF2}{MW}_{BeF2}+\left(1-x_{BeF2}\right){MW}_{NaF}}{x_{BeF2}\left(\frac{{MW}_{BeF2}}{-0.0003\times T+2.17}\right)+\left(1-x_{BeF2}\right)\left(\frac{{MW}_{NaF}}{-0.0006\times T+2.56}\right)}$$ 11

**Figure 9:**
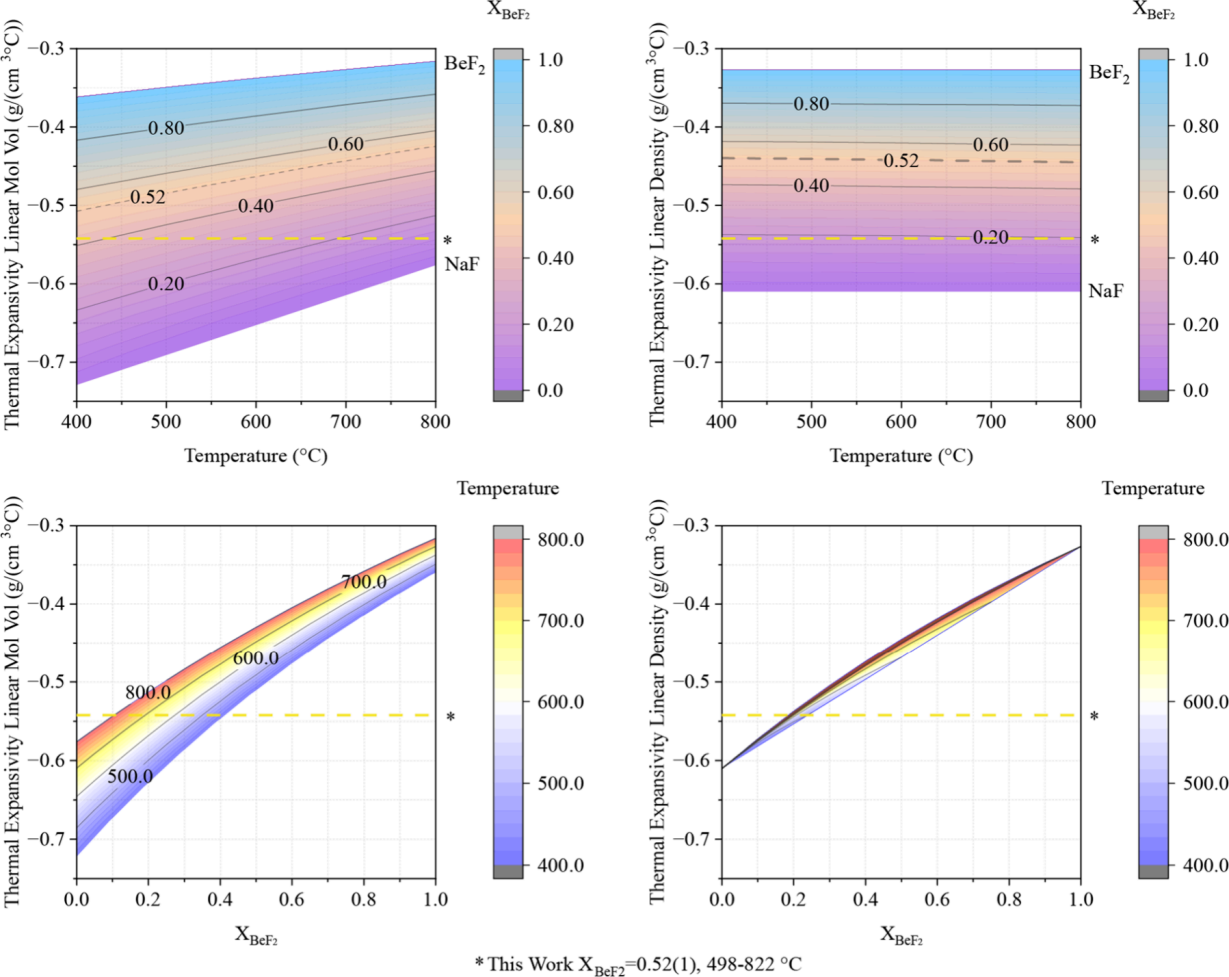
Ideal thermal expansivity of NaF-BeF_2_ as a function of the molar composition and temperature assuming (left) molar volume linearity or (right) density linearity.

Sensitivity of thermal expansivity with temperature and composition is of engineering importance and is therefore discussed here. Both eqs 8 and 9 predict a 2.5% change in thermal expansivity with ± 2 mol % deviation from 52 mol % *BeF*_2_. Equation 8 predicts a 13% change in thermal expansivity between 400 and 800 °C; eq 9 predicts a 1% change across the same temperature window; uncertainty in the experimentally measured constant slope is 4% for FNaBe and 6% for UZrFNaBe. Empirically, we would conclude that the assumption of linear density with temperature is more suitable than the linear molar volume with temperature for the single-component salts. Future structural studies should investigate the structural reason for this observed linearity.

### 4.2 Viscosity Temperature Dependence and Activation Energy Fitting

#### 4.2.1 Two Regions of FNaBe

During the temperature ramp for viscosity experiments, it was observed, using a high temperature camera located at the front of the viscometer oven, that at a specific temperature, there was a shift in the color of the salt visible between the upper and lower parallel plates as shown in Figure 10. The salt turned from a dark transparent fluid to a whitish translucent fluid. This shift can be seen in Figure 7 and Figure 8 at approximately 510–520 °C. This alteration in the color corresponded with a slight change in the trends of viscosity vs temperature and the error bars increased. Between measurements gathered from 400 to 510 °C and 520–600 °C there was a 2.4% increase in the activation energy of viscosity, as recorded in Table 2. However, since the standard deviation error among the activation energy of viscosity measurements increased over 600% between the two temperature ranges, this increase in activation energy was deemed statistically insignificant. This shift behavior was observed in all three successive runs of three separate salt samples (data provided in Supporting Information).

**Figure 10:**
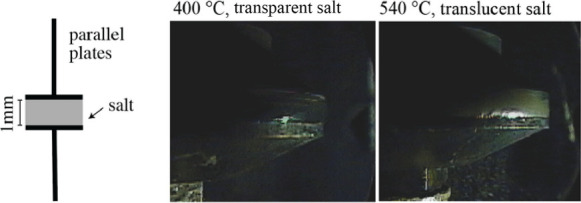
Image of FNaBe salt during parallel plate viscosity measurements at 400 and 540 °C.

#### 4.2.2 Degree of Polymerization Be vs U

A two region behavior has been previously observed in *ab initio* molecular dynamic simulations of LiF-BeF_2_ melts: at low but not dilute BeF_2_ concentration, from around 8 to 60 mol % BeF_2_, the structural relaxation time of the Be–F network indicates that the mechanism of viscosity was due to diffusion of ions, while at high BeF_2_ concentration the structural relaxation time of the network and the viscosity both increase and the viscosity scales linearly with the degree of polymerization.^4^ To test this hypothesized behavior, Figure 11 shows the activation energy of viscosity of this work compared alongside activation energy of viscosities of other fluoroberyllate melts, extracted from digitized studies on LiF-BeF_2_, LiF-BeF_2_–UF_4_, LiF-NaF-BeF_2_ titrations with two different x axes.^8,10,31,35,36^

**Figure 11:**
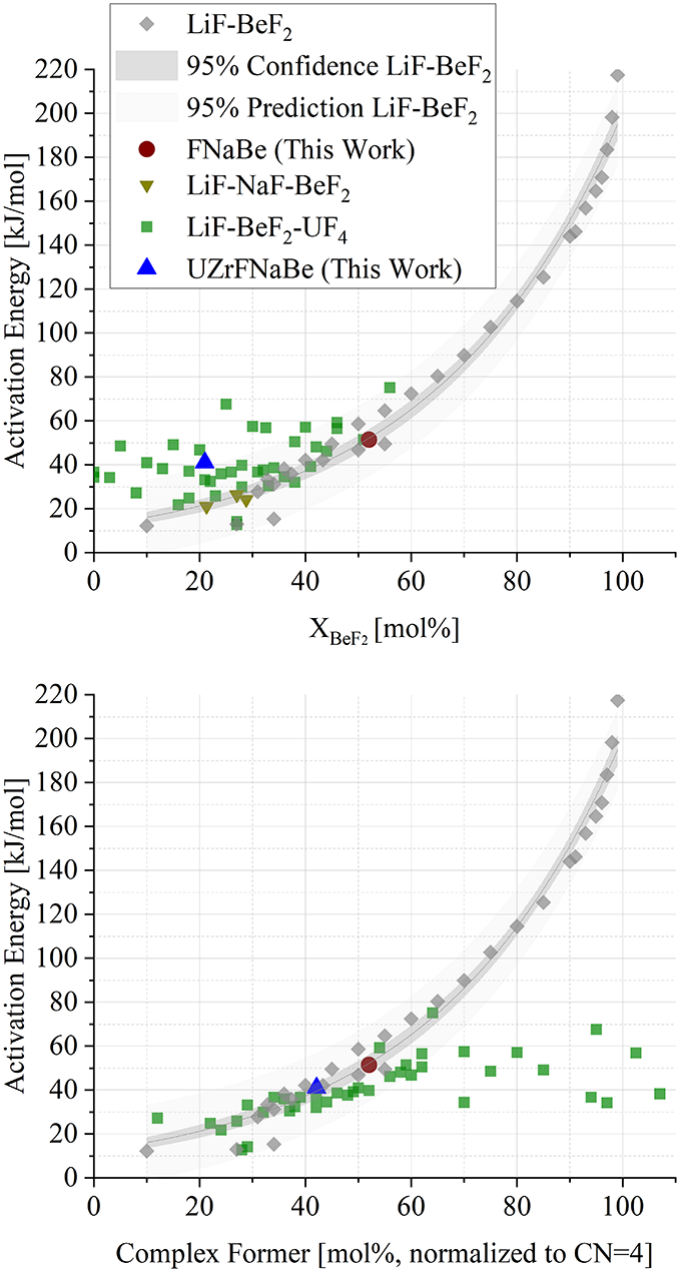
Activation energies of viscosity for FNaBe and UZrFNaBe compared to prior literature for activation energies measured from titrations of LiF-BeF_2_, LiF-BeF_2_–UF_4_, and LiF-NaF-BeF_2_.^8–10,31,35–37^ The logarithmic fit, confidence interval, and prediction interval of LiF-BeF_2_ literature data^8^ are overlaid. Source data is available the Supplementary Data file.

The activation energies of viscosity for FNaBe measured in this work fits the trend of the LiF-BeF_2_ data compiled and gathered by ref 8. at the same molar concentration of BeF_2_. Thus, at this particular fluoroberyllate concentration studied, an effect of the nature of the weakly coordinating cation (Li^+^ vs Na^+^) on the activation energy is not observed: the 7(9)% difference in activation energy is not statistically distinguishable from zero.^38^ This reinforces the modeling observations that there exists a concentration regime where the viscosity of the melt is decoupled from the diffusive motion of the noncomplexing cation, and the structural relaxation processes are dominated by the fluoroberyllate structures.^4^ Data from a study on three LiF-NaF-BeF_2_ melts is also included in Figure 11. This study is different from the other salts measured in this work and the literature that are shown in Figure 11 in that it contains two weakly coordinating cations. Further viscosity studies of the activation energy of various NaF-BeF_2_ and NaF-LiF-BeF_2_ titrations are necessary to fully determine whether this effect remains present across the compositional space.

To better compare the UZrFNaBe salt with this correlation, the uranium fluoride content was normalized to BeF_2_ in the form a term called “molar percent complex former”, where the concentration of complex forming species was counted on the basis of coordination with 4 fluoride ions. As molten U^4+^ has a U–F coordination number of 8 and Be^2+^ has a Be–F coordination number of 4, one UF_8_^4–^ is counted as equivalent to two BeF_4_^2–^ ions. The Zr^4+^ complex former content was calculated for coordination numbers ranging from 6 to 9 and the uncertainty included as *x*-axis error.^15,16^ Using this method, the activation of energy of viscosity of UZrFNaBe measured in this work here is compared to FNaBe and the LiF-BeF_2_ and LiF-NaF-BeF_2_ data in Figure 11 and found to follow the same trend with increasing complex former content, indicating that at this composition, the complex forming behavior of UF_4_ results in similar effects as BeF_2_.^8^ Furthermore, data on LiF-BeF_2_–UF_4_ titrations from ref 31 (digitized and fit to eq 5) also follow the LiF-BeF_2_ trend, with a deviation of less than 1(4)% when logarithmically fit up to 60 mol % complex former.

However, as the UF_4_ concentration in the melts increased and BeF_2_ concentration decreased there is a notable decrease in the slope of the activation energy trend when compared to lower molar percentage complex former content.^8,35^ Based on the simulation cited in ref 4, the viscosity measurements in this work, and the LiF-BeF_2_ and LiF-BeF_2_–UF_4_ titrations viscosity measurements, it can be hypothesized that at lower molar percentage complex former concentration, the small ionic species fragments such as UF_8_^4–^, BeF_4_^2–^, and Be_2_F_7_^3–^ are the dominant mechanism of viscosity through ion diffusion.^4,8,35^ Meanwhile, at high molar percentage complex former content, the behavior differs. For BeF_2_, as the concentration increases, the activation energy of viscosity increases logarithmically due to the increase in the presence of long polymeric chains. Alternatively, UF_4_ does not have the same viscosity behavior at high compound concentration, implying that while it is also a network forming compound, UF_4_ does not have the same degree of polymerization as BeF_2_.

In fuel solvents such as UZrFNaBe, potentially soluble fission products such as YF_3_, LaF_3_, CeF_3_, SmF_3_, have been estimated to be present in concentrations ranging from 2 to 4% each.^39^ The addition of these species, with varying cation coordination numbers, have been noted to affect the structure and thermophysical properties of fluoride salts.^10^ This work and its observation of previous literature has demonstrated that for the two complex forming species UF_4_ and BeF_2_, in concentrations less than around 60 mol % complex former, the slope of the activation energy of viscosity vs composition remains constant. To confirm if this behavior remains present in salt in reactor conditions with solvated fission products, in addition to viscosity experiments with added fission products to measure the changes in the activation energy, calculated coordination numbers, confirmed by neutron or X-ray diffraction are necessary.^11^ Potential methods to interpret the mechanisms behind the different polymerization behavior at high complex former content present in these molten salts include ab initio molecular dynamic simulations to observe the behavior of various sized oligomer chains; high temperature NMR^40^ or other spectroscopic methods to verify the predicted degree of polymerization, and oscillatory viscoelasticity studies to extract the complex shear modulus and measure the polymer cross-linking behavior in molten salts.^4,11,41–43^

## 5 Conclusion

Density and viscosity and their temperature dependence were measured on two fluoroberyllate salts of relevance to molten salt reactor applications, and the connection between the molecular structure of complexing ions and the observed thermophysical properties was discussed.

The equations describing the density as a function of temperature for 52:48 NaF-BeF_2_ and 70:18:12 NaF-BeF_2_–UF_4_ were calculated from experimental hydrostatic density measurements:

12 ρFNaBe[kgm3]=2454(13)·(1−0.0224(8)[%C°]·T[C°]),xBeF2=0.52(1),372to820C°

13 ρUZrFNaBe[kgm3]=3670(30)·(1−0.0226(14)[%C°]·T[C°]),xBeF2=0.21(1)xNaF=0.68(3)xUF4=0.102(5)xZrF4=0.0044(2),488to820C°

The error on density was under 0.5%, with the dominant contributions from the volume of stainless-steel bobber at temperature. Within the temperature range of measurements, the thermal expansivity was constant to within 6% uncertainty. Consequently, for ideal mixture density predictions of these multicomponent melts, empirically, we conclude that the assumption of linear density with temperature is more suitable than linear molar volume with temperature for the single-component salts. Future structural studies should investigate the structural reason behind this observed linearity.

The viscosity of the same salt samples was measured using a parallel plate rotational rheometer:

14 μFNaBe[mPa·s]=0.0103(8)e51.5(5)[kJ/mol]/RT[C°];⁣for400to600C°,andshearrate50s−1

15 μUZrFNaBe[mPa·s]=0.05(2)e41(2)[kJ/mol]/RT[C°];⁣for490to600C°,andshearrate50s−1

Together with comparisons to prior literature measurements of titrations of LiF-BeF_2_ and LiF-BeF_2_–UF_4_, it was hypothesized that, for the particular concentrations measured here, the diffusion of the weakly coordinating cations (Na^+^ or Li^+^) did not dominate the mechanism of viscosity in the presence of larger UF_8_^4–^, BeF_4_^2–^, and Be_2_F_7_^3–^ ionic complexes. Using the UZrFNaBe, FNaBe, and LiF-BeF_2_ and LiF-BeF_2_–UF_4_ titrations, two regions of BeF_2_ and UF_4_ behavior were identified; a lower complex-former concentration region where the diffusion of the aforementioned ionic fragments is the dominant mechanism of viscosity and behavior is similar between UF_4_ and BeF_2_ (normalized for coordination number), and a higher network-former concentration region where the behavior differs between UF_4_ and BeF_2_.

## Supplementary Material



## Notes

Raw mass difference density values of LiF-BeF_2,_ NaF-BeF_2_ and NaF-BeF_2_–UF_4_. partial differential equation for thermal expansivity as a function of temperature and composition based on the molar volume additivity ideal density model (density data, CSV), instrument collected viscosity data of LiF-BeF_2,_ NaF-BeF_2_, and NaF-BeF_2_–UF_4_ (viscosity data, CSV) (ZIP)
